# Biomechanical and cellular assessment of novel partially demineralized allogeneic bone plates: an ex-vivo and in-vitro study

**DOI:** 10.1186/s40729-025-00625-7

**Published:** 2025-05-20

**Authors:** Philipp Becker, Andreas Pabst, Diana Heimes, Nadine Wiesmann-Imilowski, Sven Schumann, Peer W. Kämmerer

**Affiliations:** 1 Department of Oral and Maxillofacial Surgery, University Medical Center Mainz, Augustusplatz 2, 55131 Mainz, Germany; 2 Department of Oral and Maxillofacial Surgery, German Armed Forces Central Hospital, Rübenacherstr. 170, 56072 Koblenz, Germany; 3 Department of Otorhinolaryngology, University Medical Center Mainz, Langenbeckstr. 1, 55131 Mainz, Germany; 4 Institute of Anatomy, Brandenburg Medical School Theodor Fontane, 16816 Neuruppin, Germany; 5 Institute of Anatomy, University Medical Center of the Johannes Gutenberg-University Mainz, Mainz, Germany

**Keywords:** Alveolar ridge augmentation, Allografts, Biomechanics, Scanning electron microscopy, Cell morphology

## Abstract

**Purpose:**

This study aimed to compare commercial allogeneic cortical bone plates (cCP) with innovative, differently demineralized CP (dCP) in biomechanics and human osteoblast (HOB) viability ex-vivo and in-vitro.

**Methods:**

Breaking strength (BS; in N) and flexibility (F; in mm) of cCP and dCP were assessed and compared using four groups ((1) non-hydrated, (2) hydrated for 10, (3) 30, and (4) 60 min in saline), respectively. Cell viability of HOB was evaluated by resazurin reduction on non-hydrated cCP and dCP after 3, 7, and 10 days. Scanning electron microscopy (SEM) visualized CP breaking edges, internal structures, HOB cell morphology, and growth patterns.

**Results:**

BS of hydrated dCP (d10: 15.45 ± 7.01 N, d30: 19.40 ± 3.78 N, d60: 20.31 ± 4.90 N) was significantly lower than that of non-hydrated dCP (d0: 74.70 ± 29.48 N) and native and hydrated cCP (c0: 75.00 ± 19.27 N, c10: 83.73 ± 10.92 N, c30: 83.80 ± 22.63 N, c60: 75.58 ± 14.25 N, *p* < 0.001 each). Next, dCP groups (d0: 2.64 ± 0.78 mm, d10: 2.14 ± 1.15 mm, d30: 2.76 ± 3.78 mm, d60: 2.86 ± 0.89 mm) exhibited significantly higher F than cCP groups (c0: 0.49 ± 0.14 mm, c10: 0.66 ± 0.10 mm, c30: 0.67 ± 0.16 mm, c60: 0.59 ± 0.12 mm, *p* < 0.05 each). No significant differences in F were observed among the different dCP groups. HOB cell viability was significantly increased on cCP compared to dCP after 7 (97.64 ± 2.11% vs. 76.88 ± 4.82%) and 10 days (96.14 ± 4.13% vs. 76.45 ± 4.64%; *p* < 0.001 each). SEM revealed well-defined breaking edges in cCP, whereas dCP displayed tear-off edges with shearing extensions. SEM showed disordered growth patterns and a physiological HOB cell morphology on dCP, contrasting with a parallel growth of fibroblast-like-looking HOB on cCP.

**Conclusions:**

Compared to cCP, dCP showed increased flexibility but lower breaking strength and reduced HOB vitality. The increased flexibility and a decrease in breaking strength are likely due to differences in elasticity between dCP and cCP. The use of dCP may improve clinical handling efficiency.

## Background

Alveolar ridge defects can result from tooth extraction, trauma, or periodontal disease. Reconstruction of these defects is crucial before implant placement to ensure stable primary implant fixation, achieve optimal prosthetic positioning, and maintain overall functional outcomes [1, 2]. Various surgical procedures, such as sinus floor elevation, guided bone regeneration, onlay grafting, and ridge splitting, have been reported depending on the defect type, size, and location. Both autogenous bone grafts and various types of bone substitutes, such as allogeneic, xenogeneic, and alloplastic BS, were employed based on the specific requirements of the case [2–6]. The shell technique, as proposed by Khoury, is a well-established method for alveolar ridge regeneration. In this technique, an autogenous cortical bone plate is harvested and securely fixed on the alveolar ridge defect's vestibular and lingual/palatal sides. The space between the bone plate and the defect is filled with autogenous bone particles. Shell technique allows predictable bone regeneration, addressing the height and width of the alveolar defect [7–10]. Although the shell technique usually results in high patient satisfaction [11, 12], it can also cause discomfort and donor-site morbidity [13]. Patients undergoing autogenous graft procedures may experience postoperative complications such as pain, swelling, wound healing disorders, infections, and neurosensory deficiencies [14–19]. In a study by Khoury et al. involving over 3,300 patients and over 3,800 autogenous bone blocks harvested from the mandibular angle, the inferior alveolar nerve was uncovered in approximately 4.33% of the cases, causing sensory disturbances that continued for up to six months within the nerve’s innervation area. In 0.5% of cases, hyp- or paresthesia continued for up to twelve months [20]. Additionally, obtaining autogenous grafts can extend the length of the surgery, potentially increasing the overall costs of the procedure [21–24]. Otherwise, commercially available allogeneic cortical bone plates (cCP) offer a promising alternative with no differences in complication rates and implants’ survival, even if long-term data (> 4 years) are lacking [4, 10, 19, 24–26]. While cCP eliminate donor-side morbidity, meticulous processing of allogeneic bone grafts is imperative to mitigate the risk of transmitting infectious diseases. This safeguard can be achieved through multi-step processing, including various preservation and disinfection steps such as diploidization, osmotic and oxidative treatment, solvent dehydration, gamma-irradiation, chemical processing or cleaning, lyophilization, and freeze-drying [27]. Henceforth, processed allogeneic bone compositions, readily available commercially, are deemed safe and have garnered approval for utilization [28–30]. However, due to their multi-step processing and dehydration, cCP are more brittle when compared to autogenous bone grafts and, therefore, more prone to the risk of breakage. Plate fractures can lead to complications intraoperatively and during the healing process, such as plate instability, subsequent dehiscence, or an absence of bone remodeling. A recent investigation demonstrated that cCP's breaking strength (BS) and flexibility (F) can be improved through rehydration, leading to collagen fibers' concomitant expansion in volume and flexibility [31]. Unfortunately, the impact of rehydration and the improvement of BS and F after 10 min of rehydration is limited and cannot be further enhanced beyond this point [31]. Adjusting the degree of mineralization in CP could present an additional potential strategy for refining BS and F, further optimizing their biomechanical characteristics to meet clinical requirements. Next, the biocompatibility of biomaterials and bone substitutes is essential to guarantee successful alveolar ridge augmentation, mainly due to their acellularity and avascularity. Processed allogeneic bone grafts have been rigorously evaluated in-vitro, with studies confirming their favorable biocompatibility [32, 33]. Therefore, biocompatibility is essential in assessing innovative BS and analyzing c and dCPs’ biocompatibility as the next relevant step.

This study aimed to perform a biomechanical analysis of BS and F in innovative, differentially demineralized CP (dCP) under both native and rehydrated conditions, compared to cCP in analogous states, encompassing various demineralization levels ex-vivo. Additionally, the study aimed to identify microstructural causes influencing these mechanical properties and assess biocompatibility by evaluating cell viability and morphology in human osteoblasts cultured on cCP and dCP in-vitro.

## Methods

### Biomechanical characterization

Commercial cortical bone plates (cCP) (maxgraft® cortico, botiss biomaterials GmbH, Zossen, Germany; manufactured and processed by Cells + Tissuebank Austria gemeinnützige GmbH, Krems an der Donau, Austria) and demineralized cortical bone plates (dCP) (Cells + Tissuebank Austria) with dimensions of 25 × 10 x 1 mm were employed. Both types of CP were processed by the same manufacturer (Cells + Tissuebank Austria) using the AlloTec® cleaning process [34, 35]. According to the manufacturer, the proportion of inorganic minerals in dCP was reduced by 50% compared to that of cCP. This is achieved through additional rehydration followed by acid treatment after AlloTec® processing.

For biomechanical testing, c and dCP (n = 24 each) were randomly assigned to four experimental groups (n = 6 each). These groups, labeled c0, c10, c30, c60 for cCP, and d0, d10, d30, d60 for dCP, underwent rehydration for 10, 30, and 60 min in 0.9% saline solution at room temperature. Non-rehydrated cCP and dCP served as controls (n = 6 each).

Biomechanical testing was conducted using a universal material testing machine (Model 5942; software Blue Hill, version 2.25; Instron, Pfungstadt, Germany). The testing apparatus comprised a stainless-steel sample table with a circular recess and a stainless-steel test stamp with an attached sphere. Depending on the assigned group, c and dCP were positioned either in a non-hydrated state or immediately after rehydration in an identical position over the circular cut-out. They were secured with a corresponding stainless-steel plate and four screws under precise torque wrench control to prevent asymmetrical upward movement and uneven force distribution. A uniaxial force was applied from above through the test stamp and sphere to the fixed CP at a constant strain rate of 0.5 mm/s under displacement control until CP breakage. The digital recording included breaking strength (BS, [Newton, N]) and flexibility (F, [mm]), representing the force required for breakage and the distance of deflection to the breakpoint, respectively [31].

### Cell culture

In the cell culture experiments, c and dCP with dimensions of 10 × 10 x 1 mm (n = 24 each) were randomly assigned to three experimental groups (3, 7, and 10 days; n = 8 each). Initially, each CP underwent incubation in 2 mL of osteoblast growth medium (PromoCell GmbH, Heidelberg, Germany) within standard 12-well plates (Thermo Fisher Scientific, Waltham, MA, USA) for 2.5 h at 37 °C and 5% CO_2_. Subsequently, 2 mL of medium in each well was refreshed, and human osteoblasts (HOB; PromoCell; 9.25 × 10^4^ cells/mL) at passage 3 were seeded per well. The medium was renewed every 48 h. Depending on the group assignment, after 3, 7, or 10 days, CPs were transferred to fresh 12-well plates for measurements, ensuring that only HOB attached to the CPs were included in the viability assay, excluding those that grew exclusively on the surfaces of the well plates or in the medium. For the viability assay, 0.9 mL of cell culture medium and 0.1 mL of resazurin (alamarBlue™; Invitrogen™, Waltham, MA, USA) were added per well. For the measurements, 0.1 mL of the solution was transferred from each well into a 96-well plate after three hours of incubation. The absorbance was then determined at 570 nm and 600 nm using a microplate reader (Infinite® 200 PRO, Tecan Trading AG, Männedorf, Switzerland). The percentage of resazurin reduction (%) was calculated from the measured absorption values according to the alamarBlue™ assay user manuals.

### Scanning electron microscopy (SEM)

Samples from biomechanical testing and cell culture were further analyzed by scanning electron microscopy (SEM). The samples were dehydrated, mounted on sample plates, and subjected to gold-sputtering in an argon atmosphere using an SCD 040 sputter-coater (BAL-TEC AG, Leica, Wetzlar, Germany). The visualization was conducted with a scanning electron microscope (Philips XL30, Eindhoven, Netherlands). SEM was utilized to examine the characteristic architectural bone structure within the CP following biomechanical testing. Special attention was given to the fracture edges and internal architecture to identify potential structural differences between dCP and cCP (n = 3 each) [31]. Rehydrated dCP and cCP specimens (rehydration for 10 min) were analyzed via SEM after biomechanical testing (also n = 3 each). To achieve a detailed, high-resolution examination of cell morphology and growth patterns of human osteoblasts, cCP and dCP samples from each experimental cell culture group (3, 7, and 10 days; n = 3 per group) were analyzed using SEM following the respective culture periods of 3, 7, or 10 days. As with previous assessments, the evaluation was performed exclusively in a descriptive manner.

### Statistics

The principal parameters assessed through biomechanical characterization included breaking strength (BS, [N]) and flexibility (F, [mm]). The primary parameter determined through cell viability analysis was the percentage of resazurin reduction (%). Raw data sets were documented in Excel® sheets (Microsoft Corporation, Redmond, USA) and subsequently imported into SPSS Statistics® (version 27.0; SPSS Inc., Chicago, IL, USA). The data underwent a normal distribution check using the Shapiro–Wilk test. Univariate ANOVA was employed for comparisons involving more than two independent samples, with Bonferroni and Scheffé post hoc tests for normally distributed values. In instances of non-normal distribution, the Mann–Whitney-U-Test was utilized to compare two independent groups. More than two groups were compared using the Kruskal–Wallis rank sum with the Dunn-Bonferroni post hoc test. Statistical significance was considered for *p*-values ≤ 0.05.

## Results

### Biomechanical characterization

#### Maximum breaking strength (BS)

**Fig. 1:**
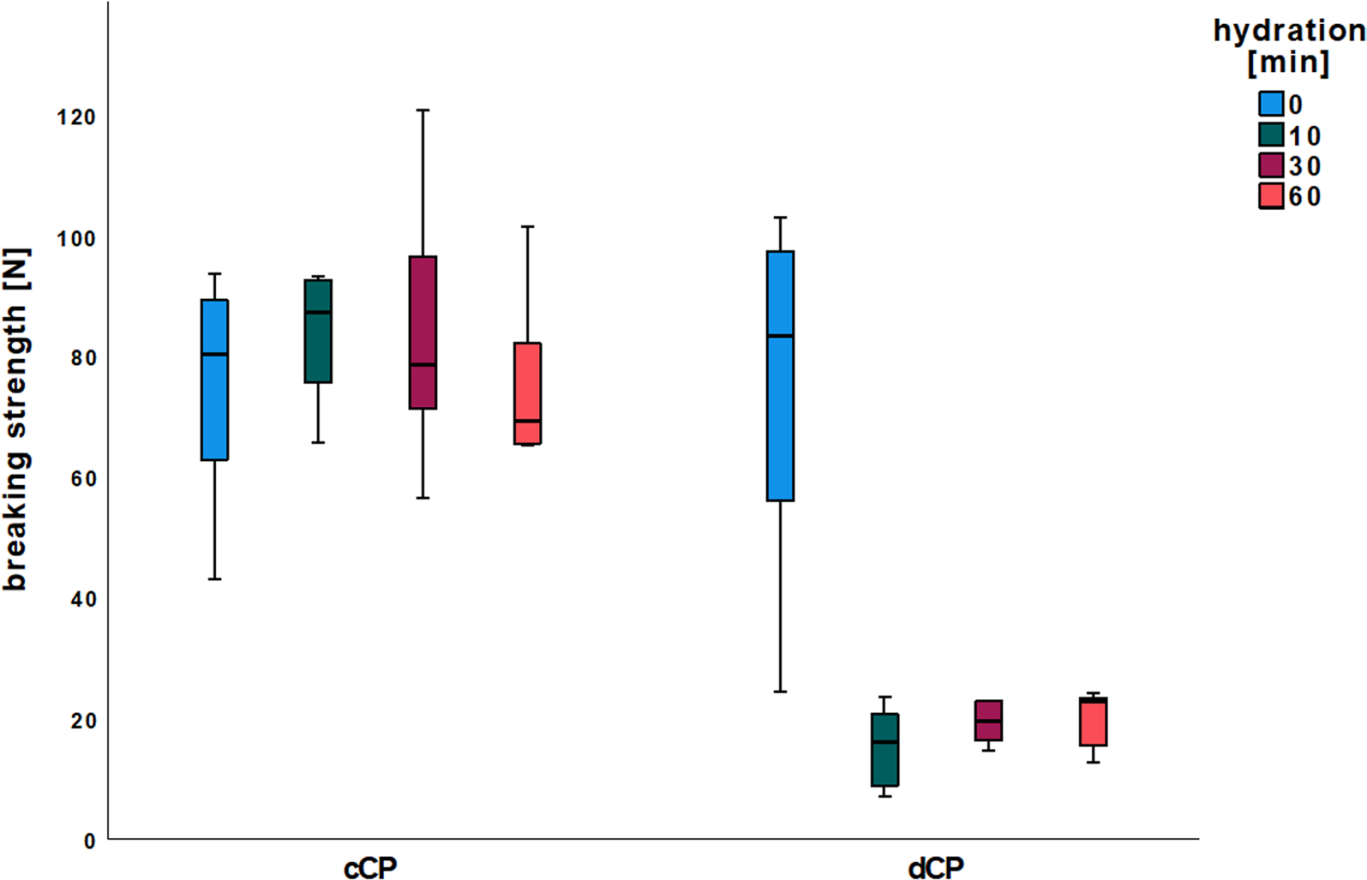
Maximum breaking strength. X-axis: commercial and demineralized CP with rehydration times ranging from 0 to 60 min. Y-axis: breaking strength [N]

No significant differences were observed between the groups of cCP dependent on the rehydration time in terms of maximum breaking strength (c0, c10, c30, c60; *p* > 0.05 each). Group d0 (74.70 ± 29.48 N) also exhibited no significant difference from groups c0 (75.00 ± 19.27 N, *p* > 0.05), c10 (83.73 ± 10.92 N, *p* > 0.05), c30 (83.80 ± 22.63 N, *p* > 0.05), and c60 (75.58 ± 14.25 N, *p* > 0.05). In contrast, the BS of all rehydrated dCP (d10: 15.45 ± 7.01 N, d30: 19.40 ± 3.78 N, d60: 20.31 ± 4.90 N) was significantly reduced compared to the groups of cCP and the group of non-rehydrated dCP (c0, c10, c30, c60, d0; *p* < 0.001 each) (Fig. 1; Table 1).

**Table 1:** Descriptive statistics of biomechanical analysis

	Breaking strength [N]		Flexibility [mm]		
Group	Mean	± SD	Mean	± SD	n
c0	75.00	19.27	0.49	0.14	6
c10	83.73	10.92	0.66	0.10	6
c30	83.80	22.63	0.67	0.16	6
c60	75.58	14.25	0.59	0.12	6
d0	74.70	29.48	2.64	0.78	6
d10	15.45	7.01	2.14	1.15	6
d30	19.40	3.78	2.76	0.70	6
d60	20.31	4.90	2.86	0.89	6

#### Flexibility

**Fig. 2:**
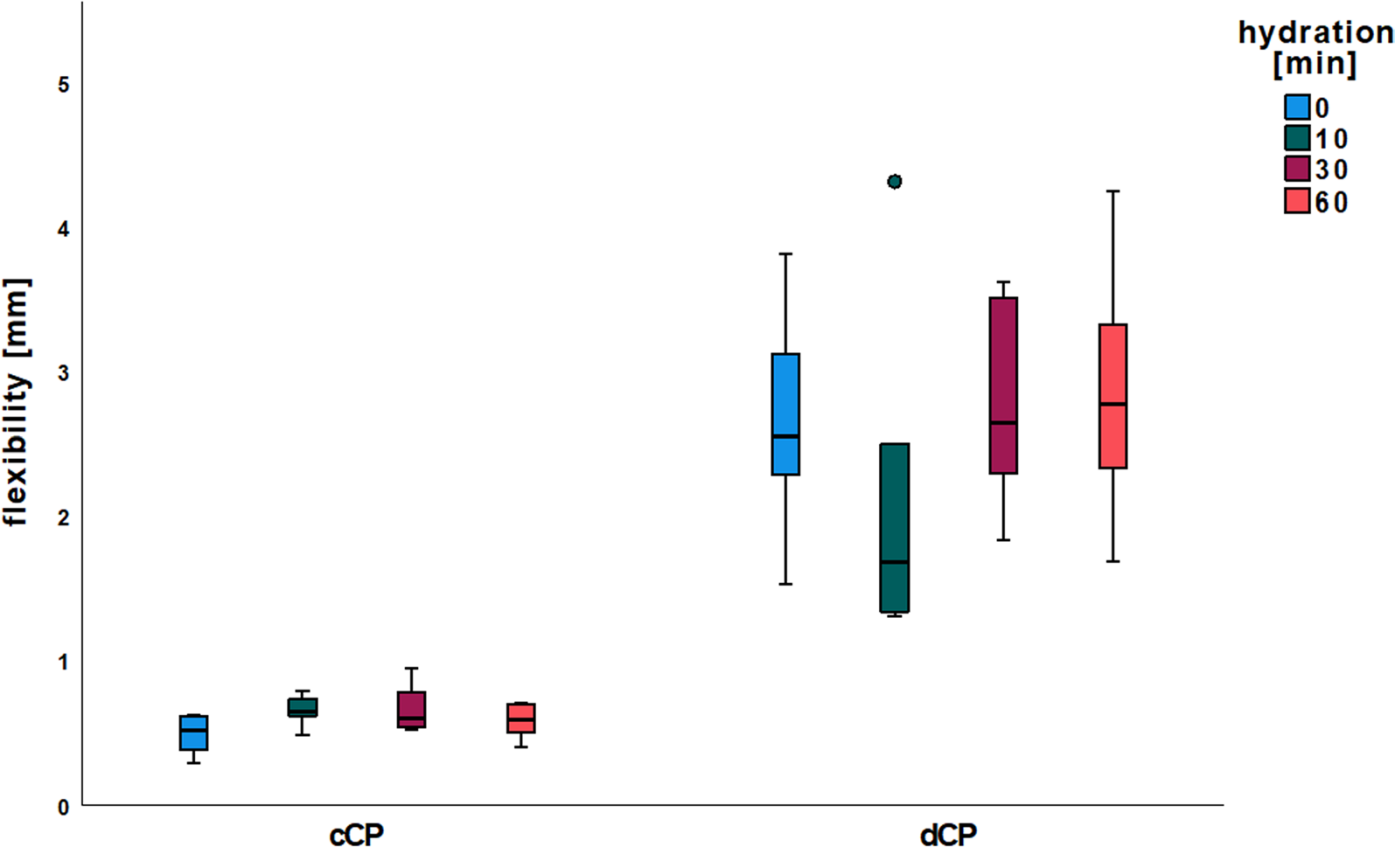
Flexibility. X-axis: commercial and demineralized CP with rehydration times ranging from 0 to 60 min. Y-axis: flexibility [mm]

Flexibility did not exhibit significant differences between the groups of cCP (c0: 0.49 ± 0.14 mm, c10: 0.66 ± 0.10 mm, c30: 0.67 ± 0.16 mm, c60: 0.59 ± 0.12 mm; *p* > 0.05 each). Similarly, there were no significant differences observed between the various groups of dCP (d0: 2.64 ± 0.78 mm, d10: 2.14 ± 1.15 mm, d30: 2.76 ± 3.78 mm, d60: 2.86 ± 0.89 mm; *p* > 0.05 each). Nevertheless, all groups of dCP (d0, d10, d30, d60) displayed a significantly increased flexibility compared to the groups of cCP (c0, c10, c30, c60; *p* < 0.05 each) (Fig. 2, Table 1).

### Cell viability

**Fig. 3:**
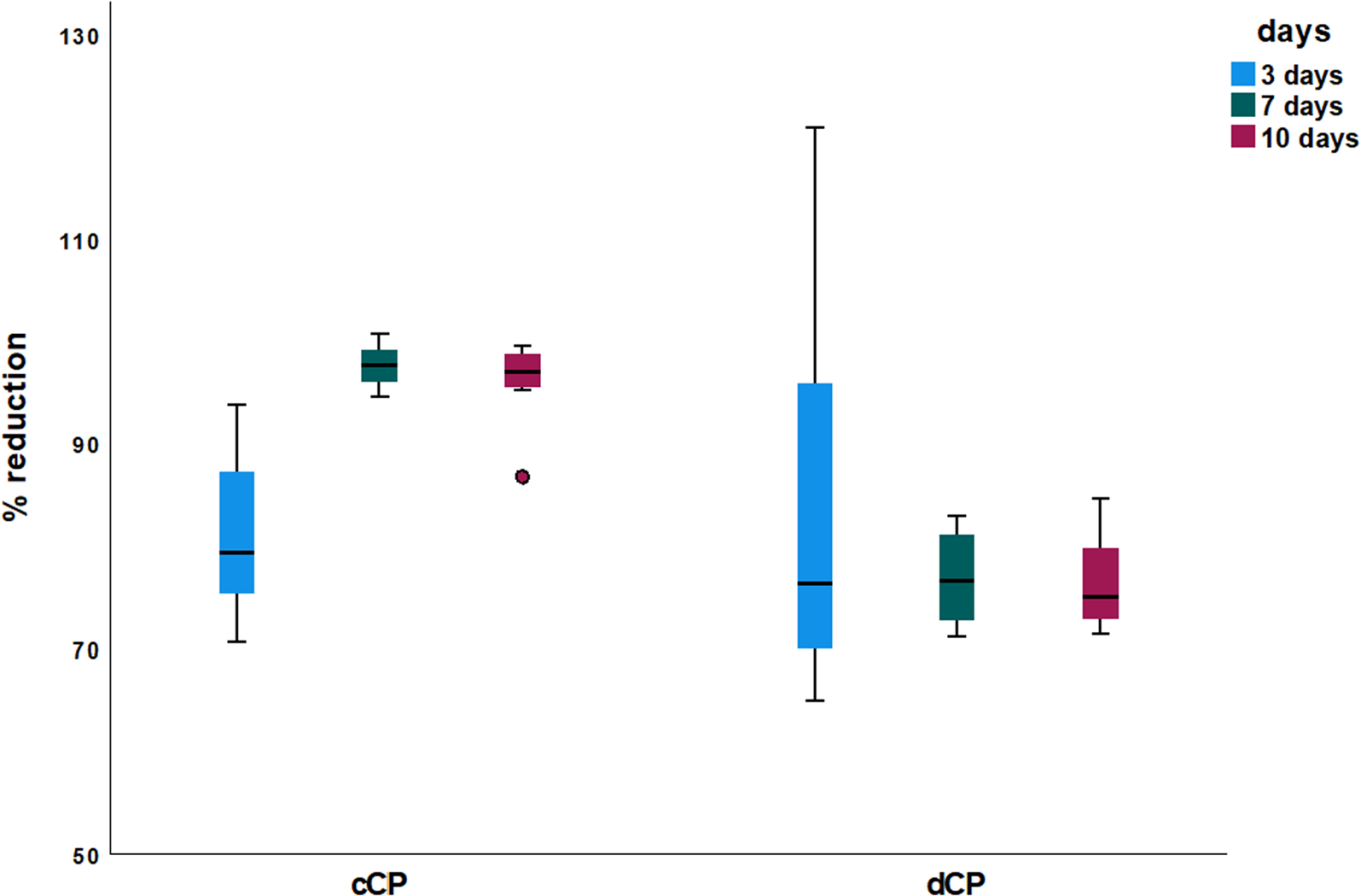
Cell viability measurements after 3 h of resazurin incubation. X-axis: experimental groups (3, 7, and 10 days) of commercial and demineralized CP. Y-axis: percentage of resazurin reduction (%)

In the absorption measurements after resazurin incubation, a significantly higher resazurin reduction was measured in the cCP group at the test time points 7 days (97.64 ± 2.11% vs. 76.88 ± 4.82%; *p* < 0.001) and 10 days (96.14 ± 4.13% vs. 76.45 ± 4.64%; *p* < 0.001) compared to the demineralized group. However, at the test time point after 3 days, there were no significant differences between cCP and dCP (81.07 ± 8.47% vs. 83.83 ± 21.02%; *p* > 0.05 each). After resazurin incubation, there were no significant differences within the dCP group between the different test time points after 3, 7, and 10 days (*p* > 0.05 each). In contrast, within the cCP group, the resazurin reduction at the test time point after 3 days was significantly lower than at the test time points after 7 and 10 days (*p* < 0.05 each); there were no significant differences between the test time points after 7 and 10 days (*p* > 0.05) (Fig. 3, Table 2).

**Table 2:** Descriptive statistics of cell viability analysis

		resazurin reduction [%]		
Group	Days	Mean	± SD	n
commercial	3	81.07	8.47	8
	7	97.64	2.11	8
	10	96.14	4.13	8
demineralized	3	83.83	21.02	8
	7	76.88	4.82	8
	10	76.45	4.64	8

### Scanning electron microscopy

#### Breaking edges and internal structure

**Fig. 4:**
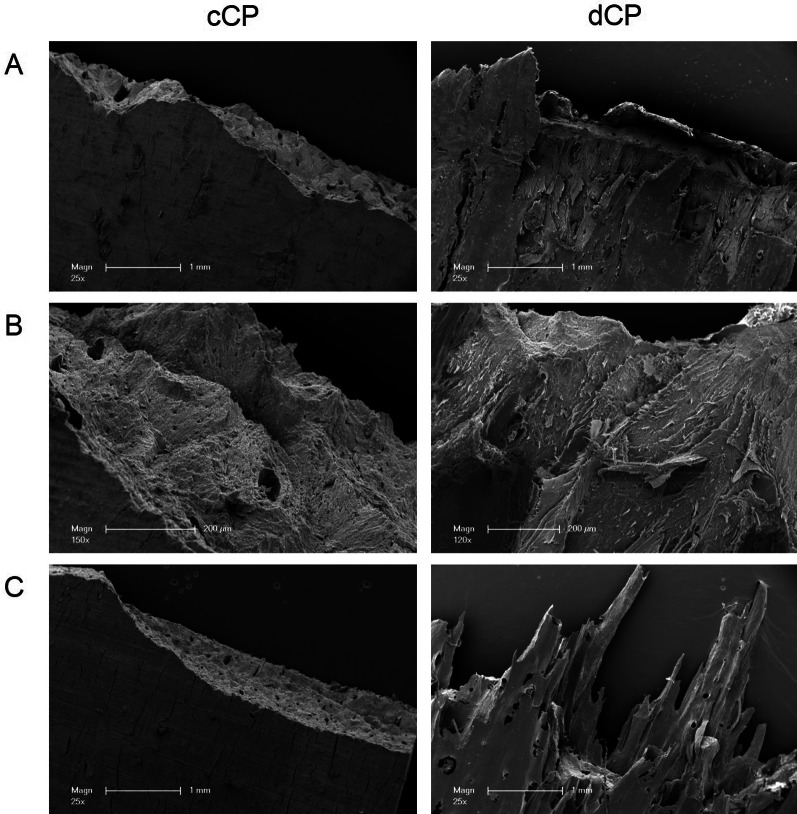
Scanning electron microscopy of CP breaking edges and internal structure. Breaking edges and internal structure of commercial and demineralized CP after biomechanical testing in the non-rehydrated state are shown in low (**A**) and intermediate magnification (**B**) and after 10 min of rehydration (**C**). Commercial CP showed clearly defined breaking edges and a rough, sand-like surface. In contrast, in demineralized CP, tear-off edges with individual elongated shearing extensions and small soft-tissue fibrous structures were visible

Observing the microstructure of the fracture surface revealed notable distinctions between cCP and dCP. In low-magnification SEM images, clear fracture sites and edges were evident in cCP, contrasting with dCP, which exhibited tear-off edges featuring elongated shearing extensions extending beyond the actual breaking edge (Fig. 4 A).

The non-hydrated cCP displayed a rough, grainy, sand-like surface at high magnification. Conversely, the fracture edge surface of the non-hydrated dCP exhibited small soft-tissue fibrous structures (Fig. 4 B).

Following 10 min of rehydration (Fig. 4 C), the fracture site of the cCP became even more sharply defined along a specific border, and the internal surface of the fracture site appeared smoother. Longitudinal microcracks formed around the cCP surface after rehydration. In comparison, the previously described tails of the dCP appeared longer and more pronounced after rehydration, with the surface structure also appearing smoother in this context.

#### Human osteoblast coverage and growth patterns on cortical plates

**Fig. 5:**
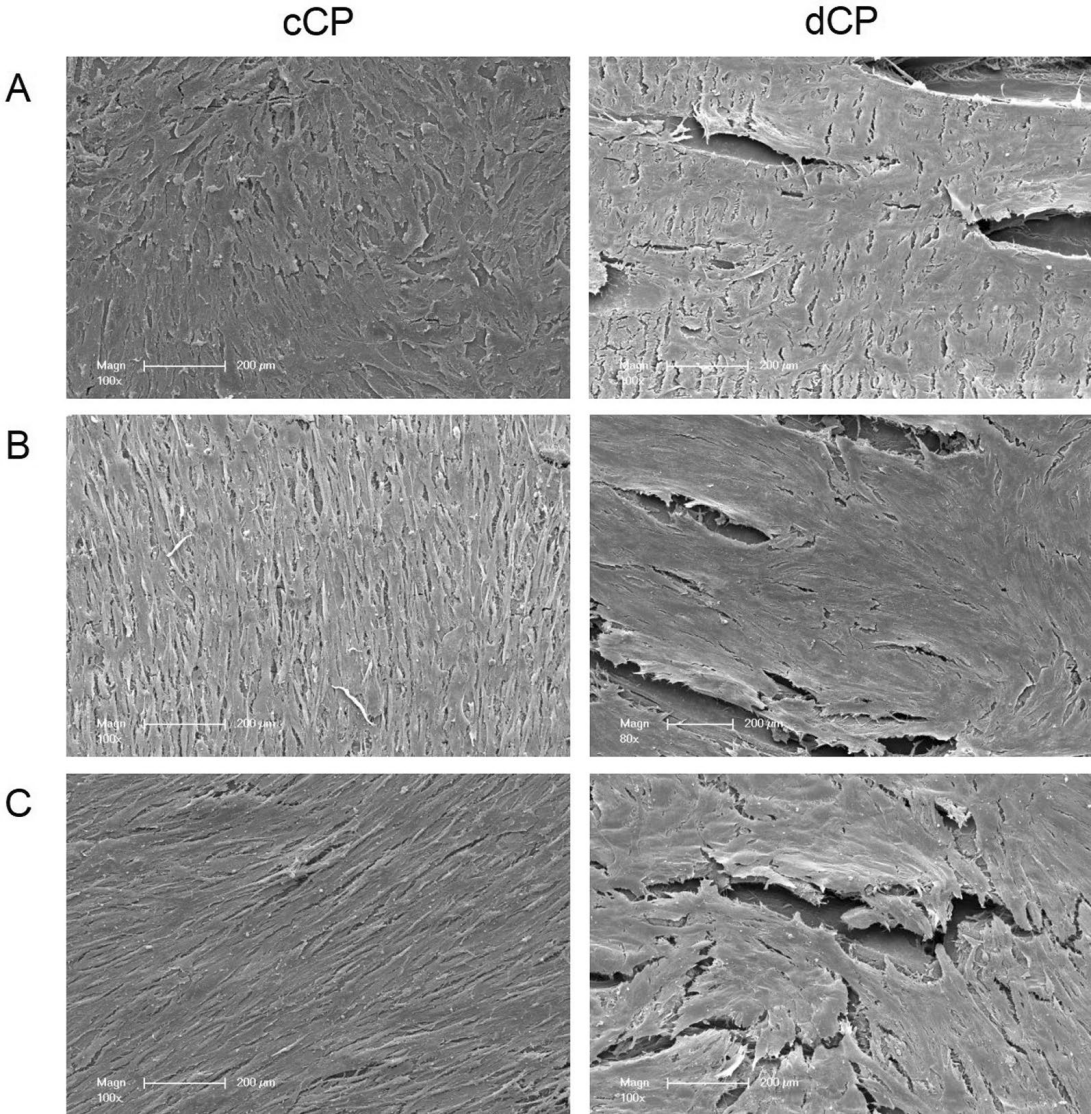
Scanning electron microscopy of HOB growth pattern. HOB cultured on commercial CP showed different growth patterns at the different time points after 3 (**A**), 7 (**B**), and 10 days (**C**) compared to the HOB seeded on demineralized CP. Commercial CP showed a parallel and more structured growth, while the three-dimensional growth patterns on demineralized CP were disordered

**Fig. 6:**
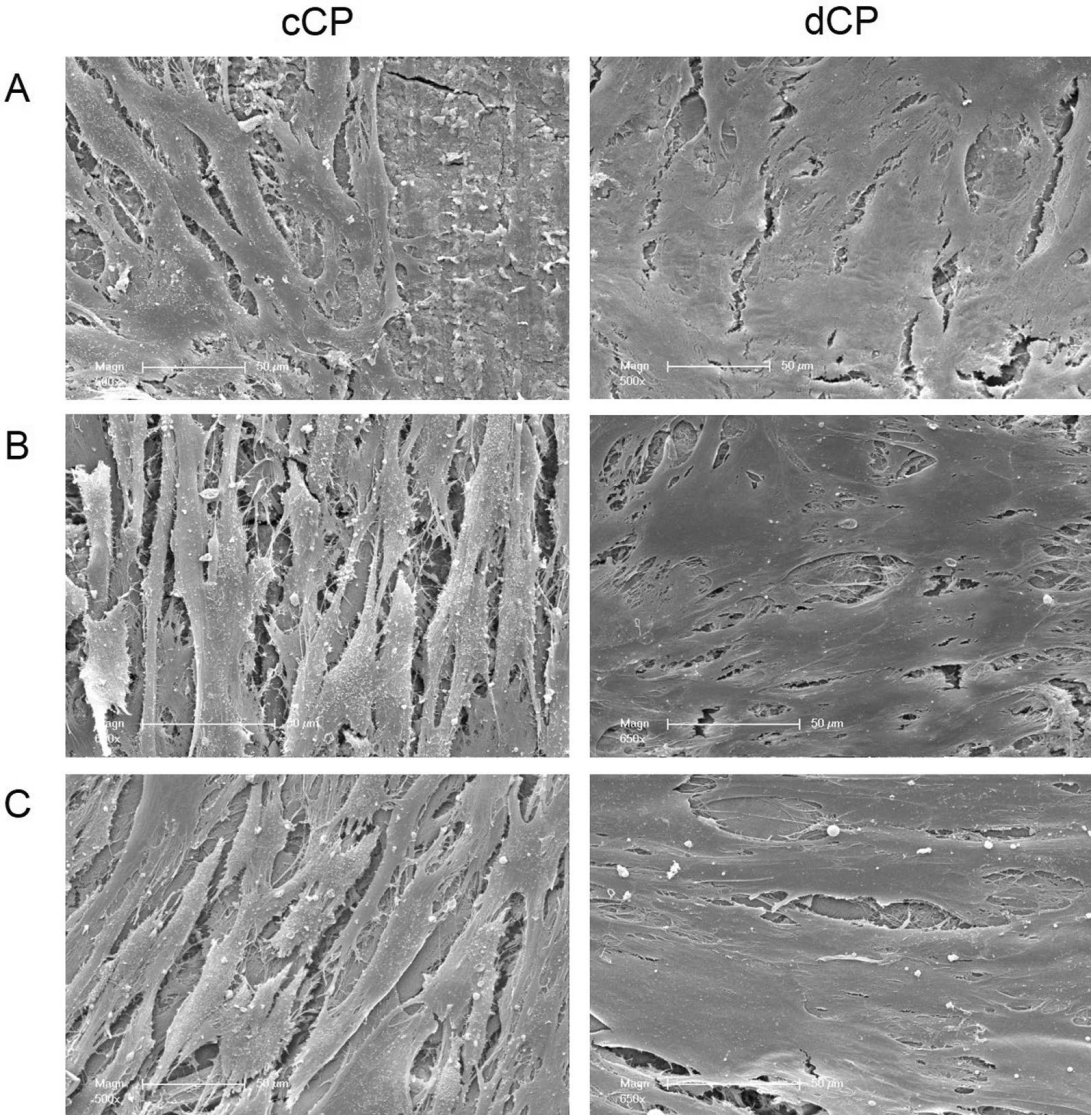
Scanning electron microscopy of CP overgrown with HOB in detail. HOB cultured on commercial CP showed a different cell morphology at the different time points after 3 (**A**), 7 (**B**), and 10 days (**C**) compared to HOB seeded on the demineralized CP. SEM illustrated a physiological polygonal HOB cell morphology on demineralized CP. In contrast, the HOB on commercial CP presented elongated and fibroblast-like in appearance

After 3 days, both cCP and dCP were nearly entirely covered by HOB. However, distinctive differences in HOB growth patterns were observed between the two CP types. After 3 days, HOB on cCP displayed an initially disordered and polygonal shape in low- and high-magnification SEM images, gradually transitioning into a more elongated form. Thin fibrous processes connected the HOB to the cCP surface, forming a monolayer, with some CP structure still visible underneath. The HOB on dCP appeared polygonal without a regular orientation. In certain areas, cell boundaries had merged, making individual cells indistinguishable, while in other regions, cells formed dense connections to neighboring cells via extensions on all sides (Fig. 5 A, 6 A).

By the 7th day, HOB on cCP aligned parallel in a specific direction, exhibiting an elongated and flat fibroblast-like shape. Though individual cells were still separated by gaps, indicative of the beginning of cell stratification, the CP surface was visible in some areas. In contrast, HOB on dCP formed a densely packed poly layer, with polygonal-shaped cells showing disorder and fusion at cell boundaries or broad connections to neighboring cells through small extensions. No significant gaps between individual cells were observed, and any large cracks within the cell layer were likely artifacts from the fixation process (Fig. 5, 6 B).

After 10 days, a distinct HOB multilayer with parallel-aligned cells had formed on the cCP. The longitudinal cell shape increased in length with decreasing width, and the gaps between individual cells decreased. However, these gaps did not merge at the borders. HOB continued to exhibit polygonal shapes without ordered cell orientation on the dCP. In some areas, the gaps between cells had completely disappeared as HOB grew overlapping, and cell boundaries appeared to merge in a spatial three-dimensional structure. In both cases, tight cell bonds led to cracks during SEM fixation, creating artifacts (Fig. 5 + 6 C).

## Discussion

The allogeneic shell technique is well-established for reconstructing alveolar ridge defects [24, 28]. It has proven effective in facilitating ample bone regeneration and achieving high bone quality. Dehiscences have been reported as the most common complication of this technique, even though later implant placement may be possible in most cases [28, 36, 37]. Next, CP can fracture during screw fixation due to its brittleness and low flexibility found in commercial allogeneic CP [31]. Efforts are being made to improve its mechanical properties to achieve better clinical results and expand its applications. In this context, rehydration can improve the ductility of dry bone allografts [38]. A recent study demonstrated an augmentation in BS and F of commercial allogeneic CP following saline rehydration [31].

This study also tentatively reproduced the absolute flexibility values (in mm) and their increase through rehydration. The results’ lack of statistical significance can be attributed to the smaller sample size of cCP per group (n = 6 vs. n = 10) and the increased number of groups (n = 8 vs. n = 4). Similarly, saline rehydration led to a trend towards increased BS in this study. However, the absolute values are higher in the current study than those reported in the previous study [31]. This difference could be due to donor bone quality variations, such as age or bone density. Another factor could be the variability introduced by the CP processing method, which might result in differing degrees of demineralization or variations in collagen content and quality. These factors could influence the efficacy of rehydration and account for the observed differences in BS and F values.

Demineralization did not negatively influence the BS of non-hydrated dCP. However, dCP rehydration resulted in a significant BS reduction. Conversely, F was enhanced by reducing the mineral content of dCP, as evidenced by a more than fivefold increase in bending distance before dCP fracture. Additional rehydration of dCP showed no discernible influence on F. Therefore, non-rehydrated dCP could be an alternative to rehydrated cCP with similar BS and a significantly increased F. According to the manufacturer's information, the commercial allogeneic CP used in this study contain approximately 30% natural collagen types I and III and approximately 5% water [31].

The manufacturer’s specifications reduced the proportion of inorganic minerals in the dCP by 50%. This reduction likely results in decreased intra-material tensions as the flexible collagen fibers absorb these tensions. BS reduction found in rehydrated dCP could be due to the collagen fibers, which are surrounded by a diminished inorganic matrix and swell significantly during rehydration. This swelling makes the fibers highly elastic, increasing F but reducing BS. After reaching the bending maximum, dCP, and even more notably, rehydrated dCP, were more prone to buckle or shear, exhibiting less clear fracture lines than cCP. A detailed examination of the microscopic breaking edge structure of dCP revealed a tear-off edge with individual elongated shear processes extending beyond the actual breaking edge. While high flexibility is desirable to prevent CP fractures, it should be carefully controlled. Excessive F during the healing phase after alveolar ridge augmentation could lead to micro-movements, potentially resulting in sequestration, failure of bony integration, and subsequent loss of augmentation.

On the other hand, the reduction in inorganic mineral content in dCP influences their mechanical properties, particularly elasticity. While a lower BS might seem disadvantageous, a combination of high elasticity (E) and F could significantly enhance the clinical performance of the CP by reducing the fracture risk. In clinical scenarios, CP are subjected to multi-axial stresses, such as bending and localized forces during drilling, rather than the uniaxial forces simulated in mechanical testing. High F allows plates to deform under stress without breaking, while increased E ensures resilience during shaping and screw insertion. These properties could make the CP less prone to fracture under surgical and postoperative conditions, even with lower BS.

Thus, the necessity for high BS in clinical applications may be reconsidered. Prioritizing F and E could better align material properties with surgical requirements and improve outcomes. Future research should focus on mimicking clinical forces to determine the optimal balance of mechanical properties for different indications.

Examining X-ray images of the surgical area after the healing phase of an augmentation done with the allogeneic shell technique, cCP can be clearly distinguished from the rest of the augmented area [7, 24, 28, 31]. At re-entry, typically 4–6 months post-augmentation, CP generally seems fixed or integrated at the osseous interface but has not converted significantly into local alveolar bone. This observation can be attributed to the dense cortical bone structure, which inherently slows remodeling [39]. However, the demineralization of CP may accelerate this process by enhancing its remodeling capacity.

Demineralization exposes a greater portion of the bone matrix (e.g., collagen). The attachment of osteoclasts to this matrix is a critical step in bone remodeling [40]. That may enhance osteoclastic recruitment and activity, accelerating resorption and subsequent replacement by local bone tissue. Additionally, the increased porosity of dCP could enhance vascular infiltration, further supporting integration and remodeling. Thus, demineralized CP may overcome the remodeling limitations of cortical bone, leading to faster conversion into local alveolar bone.

A study by Spin-Neto et al., which analyzed fresh-frozen block bone allografts in-vivo, presented findings from a patient group that was augmented using only autogenous cortical bone. After 6–8 months of healing, the results showed less than 4% of vital bone and almost 84% of necrotic bone. This suggests minimal real remodeling and revascularization of cortical bone [39]. SEM of HOB grown on cCP displayed a flattened, elongated, fibroblast-like appearance, with predominantly longitudinal connections to neighboring cells. Next, HOB exhibited a parallel orientation and two-dimensional layered growth patterns with gaps between individual cells. In contrast, HOB cell configuration and growth pattern on dCP were notably different. Here, HOB exhibited a more physiological osteoblast-like, polygonal configuration with a three-dimensional, overlapping, dense growth pattern featuring connections to neighboring cells in all spatial directions. This observation aligns with a study by Schmidt et al. exploring HOB cell morphology on various metals in-vitro. The study found that materials with a smooth surface elicited growth by flattened, elongated, and fibroblast-like HOB, while metals with a rough, sandblasted surface promoted a physiological and three-dimensional growth pattern of HOB. The researchers concluded that a rough material surface appears to mimic a more natural environment for osteoblasts and serves as a foundation for the successful osseointegration of an implant [41]. Investigations on metals cannot directly be translated to allogeneic bone compositions. HOB growth patterns on dCP appeared more physiological than those on cCP. This suggests better tissue integration and higher biocompatibility for dCP. Caution is advised at this point, as excessively demineralized bone has been criticized for having lower osteoconductive properties. To conclude, a certain middle way must be found, whereby an ideal degree of mineralization leads to maximum bone generation by optimizing the sum of osteoinduction and -conduction [42–44]. However, when considering cell viability measurements, the resazurin reduction in the cCP group was significantly increased after 7 and 10 days of cell culture compared to the dCP group. This suggests that cCP may favor HOB proliferation and viability. Moreover, resazurin reduction did not differ in the dCP group when comparing individual test time points despite clear evidence of increasing cell proliferation in SEM. This discrepancy could be because the densely packed cell layers on dCP are hindering the penetration of resazurin, reaching only the top cell layer and being reduced by a single layer of cells. Additionally, it is known that a rough material surface, conducive to physiological three-dimensional cell growth, can promote osteoblast differentiation while concurrently downregulating cell proliferation. This may explain the observed reduction in HOB proliferation in the dCP group [41]. In summary, viability measurements and SEM imaging of dCP confirmed sufficient biocompatibility. Beyond fostering more physiological osteoblast growth, dCP microstructure also offers biomechanical advantages. In its non-rehydrated state, it achieves significantly higher F while maintaining constant BS. Nevertheless, adherence to the application principles of the allogeneic shell technique is crucial. This entails extraoral trimming and drill hole preparation, stable positioning and fixation of the CP using adjusting screws, meticulous smoothing of edges, and ensuring that augmentation occurs within the skeletal envelope [28].

While this study provides valuable insights into the biomechanical and cellular characteristics of dCP and cCP, several limitations must be acknowledged. First, the study lacks long-term data on how these properties influence clinical outcomes in-vivo. Future in-vivo studies should investigate the remodeling and integration processes, such as the vascularization of dCP, using for example angiogenesis models like the chorioallantoic membrane (CAM) assay or small-animal models, followed by (immuno-)histopathological analysis or bone evaluation using micro-computed tomography [45, 46].

While the study demonstrates clear differences in HOB morphology and growth patterns between dCP and cCP, a more detailed investigation into the variability of dCP surface texture is warranted. Surface roughness and topography are known to significantly influence HOB [41]. Future research should quantitatively assess surface roughness, e.g., using atomic force microscopy (AFM) [47], to correlate specific surface characteristics with osteoblast behavior, and to compare surface roughness among individual CPs within each group (dCP or cCP). Future studies could compare cCP and dCP groups and different biofunctionalization methods, such as platelet-rich fibrin (PRF), hyaluronic acid, or enamel matrix proteins. A possible hypothesis is that the dCP structure could allow for better absorption of these substances, potentially improving biocompatibility by improved cell proliferation and differentiation.

The sample size, particularly for SEM analyses, was relatively small, limiting the ability to generalize findings about cell proliferation and morphology. Increasing the number of SEM samples in future studies would enhance the robustness of conclusions regarding osteoblast adaptation to dCP surfaces. Additionally, complementary analyses, such as immunohistochemical staining for osteogenic markers (e.g., Runx2), could provide further insight into differentiation pathways influenced by CP microstructure [48]. 

From a biomechanical perspective, the study focused on uniaxial breaking strength, whereas clinical applications involve multi-axial loading, including shear and torsional forces. Intraoperative handling steps such as shaping, drilling, and screw fixation were not simulated. Further studies should include protocols that replicate these clinical procedures to evaluate whether dCP maintains structural integrity during manipulation or presents a higher risk of intraoperative CP fractures.

Finally, while the potential clinical benefits of increased flexibility versus breaking strength were discussed, future work should explore specific surgical applications where these properties are advantageous. For example, dCP may be particularly advantageous in cases where plates must conform to complex anatomical structures, such as in bone regeneration in anatomically complex regions like the anterior mandible and maxilla, the zygomaticoalveolar crest, or the lingual and palatal area of the jaws.

Furthermore, future research should compare dCP and cCP in controlled clinical studies, assessing both their handling properties during surgery and their long-term success and survival rates.

## Conclusions

Non-rehydrated dCP showed the same BS and significantly increased F compared to rehydrated cCP, which could be due to differences in elasticity between dCP and cCP. Therefore, non-rehydrated dCP could be an alternative to rehydrated cCP. Increased F of dCP could improve adaption to different alveolar shapes and better absorption of applied forces. This can indirectly lead to higher durability and a lower risk of breaking and improve clinical handling efficiency. However, further research is needed to evaluate its biocompatibility thoroughly.

## Data Availability

No datasets were generated or analysed during the current study.
